# Premature ovarian insufficiency in patients with systemic lupus erythematosus on cyclophosphamide: a systematic review and meta-analysis

**DOI:** 10.3389/fendo.2026.1775060

**Published:** 2026-05-28

**Authors:** Manuel Ramón García-Sáenz, Claudia Ramírez-Rentería, Etual Espinosa-Cárdenas, Ernesto Sosa-Eroza, José Luis Eduardo Doval-Caballero, Paulo César Gete Palacios, Fabiola Pazos-Pérez, Mario César Ocampo-Torres, Rocío Catana-Hernández, Miguel Ángel Vázquez-Zaragoza, Adolfo Camargo-Coronel, Juan Rodrigo Gómez-Bernal, Aldo Ferreira-Hermosillo

**Affiliations:** 1 Departamento de Endocrinología, Hospital de Especialidades del Centro Médico Nacional Siglo XXI, Instituto Mexicano del Seguro Social, Mexico City, Mexico; 2 Unidad de Investigación Médica en Enfermedades Endocrinas, Hospital de Especialidades del Centro Médico Nacional Siglo XXI, Instituto Mexicano del Seguro Social, Mexico City, Mexico; 3 Obesity Clinic, Instituto Nacional de Ciencias Médicas y Nutrición “Salvador Zubirán”, Mexico City, Mexico; 4 Departamento de Nefrología, Hospital de Especialidades del Centro Médico Nacional Siglo XXI, Instituto Mexicano del Seguro Social, Mexico City, Mexico; 5 Departamento de Reumatología, Hospital de Especialidades del Centro Médico Nacional Siglo XXI, Instituto Mexicano del Seguro Social, Mexico City, Mexico; 6 Unidad de Posgrados, Facultad de Medicina, Universidad Nacional Autónoma de México, Mexico City, Mexico

**Keywords:** cyclophosphamide, fertility preservation, gonadotoxicity, lupus nephritis, meta-analysis, premature ovarian insufficiency, systematic review, systemic lupus erythematosus

## Abstract

**Background:**

Premature ovarian insufficiency (POI) is a major long-term adverse effect of cyclophosphamide (CYC) therapy in systemic lupus erythematosus (SLE). Reported prevalence varies due to differences in populations, protocols, and diagnostic criteria. Objective: To estimate the prevalence of POI in women with SLE exposed to CYC and explore sources of heterogeneity.

**Methods:**

MEDLINE, Embase, Web of Science, Scopus, and LILACS were searched from inception through October 2024. Twenty-one studies were included in the systematic review; 12 CYC-exposed cohorts contributed to the primary meta-analysis. Two reviewers independently extracted data and assessed study quality using the Joanna Briggs Institute checklist.

**Results:**

The pooled prevalence of POI in SLE treated with CYC was 15% (95% CI 7-26%), with substantial heterogeneity (I^2^ = 85%; Cochran’s Q = 73.98 p <0.0001; tau^2^ = 0.0353). In contrast, prevalence among unselected SLE populations was markedly lower, confirming the potential gonadotoxic effect of CYC. Temporal analysis showed reduced prevalence in studies published after 2005 (9%) compared with pre-2005 (29%), coinciding with adoption of Euro-Lupus low-dose protocols. Meta-regression identified age at exposure as a significant predictor (β = 0.21, 95% CI: 0.04-0.38, p = 0.017). Cumulative CYC dose was not independently associated with prevalence, likely reflecting the widespread adoption of lower-dose regimens after 2005. Methodological sensitivity analyses using logit transformation (20.0%, 95% CI 12.7-30.1) and GLMM (14.5%, 95% CI 7.1-27.2) were added to assess model robustness. Funnel-plot and the Egger’s test indicated no evidence of publication bias (p = 0.91).

**Conclusions:**

Women with SLE treated with CYC show a substantial burden of POI, with an estimated prevalence of approximately 15%. These results underscore the need for standardized diagnostic criteria, fertility counseling, and individualized treatment decisions.

**Systematic review registration:**

https://www.crd.york.ac.uk/PROSPERO/view/, identifier CRD42025641213.

## 1 Introduction

Premature Ovarian Insufficiency (POI) represents a major reproductive health concern with significant implications for women affected by systemic lupus erythematosus (SLE). While the prevalence of POI in the general population is now estimated to be approximately 1.0-3.7%, according to recent epidemiologic data (1–5), studies in women with SLE report markedly higher rates, ranging from 11% to 54% (6). This striking difference underscores the strong association between SLE and impaired ovarian reserve.

Among the iatrogenic risk factors, cyclophosphamide (CYC) remains the most important contributor to POI in patients with SLE (7–9). Multiple studies have consistently demonstrated that higher cumulative CYC doses substantially increase the risk of ovarian failure, particularly in older women and those with longer disease duration (10). For instance, Akawatcharangura et al. reported a hazard ratio of 17.0 for POI among women receiving more than 10 grams of CYC (8). Similarly, the LUMINA LVIII study highlighted that CYC use significantly increases the risk of gonadal failure, with the highest vulnerability observed in Hispanic and African American women (10). Nevertheless, not all cases of POI in SLE can be explained by CYC exposure alone. Disease activity and cumulative organ damage have also been implicated in its pathogenesis, suggesting potential autoimmune mechanisms independent of immunosuppressive therapy (11). CYC remains indicated as a steroid-sparing or rescue agent for severe, organ-threatening manifestations of SLE such as lupus nephritis and neuropsychiatric lupus (12, 13). Recent evidence indicates that high disease activity during the first three years after diagnosis may be a critical determinant for POI development, regardless of CYC treatment (7, 10). For these patients, gonadotropin-releasing hormone analogues (GnRHa) were introduced in the early 2000s as ovarian-protective co-therapy during CYC treatment, though their use remains variable worldwide (14, 15).

The clinical consequences of estrogen deficiency extend well beyond fertility, encompassing increased risks of cardiovascular disease, osteoporosis, and impaired quality of life (7, 16). In addition, POI is not defined uniformly across the literature. Some studies rely mainly on prolonged amenorrhea or premature menopause as clinical surrogates, whereas others apply stricter endocrinologic criteria requiring menstrual disturbance plus menopausal-range gonadotropins and low estradiol. Explicitly acknowledging these diagnostic yardsticks is essential, because they directly affect prevalence estimates and interpretation across rheumatology and endocrinology studies. In this context, we conducted a systematic review and meta-analysis to provide comprehensive evidence on the prevalence of POI among women with SLE, with particular emphasis on the role of CYC exposure and temporal trends following changes in treatment protocols.

## 2 Methods

This systematic review and meta-analysis were conducted following Preferred Reporting items for Systematic Reviews and Meta-Analyses (PRISMA) guidelines. The study protocol was registered in PROSPERO (CRD42025641213).

### 2.1 Eligibility criteria

Observational studies (cohort, cross-sectional, and case-control designs) reporting POI prevalence in women with SLE with CYC exposure were included. Participants were women of reproductive age diagnosed with SLE according to American College of Rheumatology (ACR) 1982/1997/2019 classification criteria, defined by the presence of ≥4 of 11 clinical and immunologic features (malar rash, discoid rash, photosensitivity, oral ulcers, arthritis, serositis, renal disorder, neurologic disorder, hematologic disorder, immunologic disorder, or positive anti-nuclear antibodies) (17–19). Although our PROSPERO protocol initially restricted inclusion to studies reporting POI defined by both amenorrhea and compatible hormonal profiles, during full-text review we found that this approach would exclude a large proportion of the available evidence. Therefore, we broadened eligibility to include all studies that explicitly reported a diagnosis of POI, regardless of whether full hormonal criteria were applied. This allowed us to capture the heterogeneity of definitions used in the field.

### 2.2 Information sources and search strategy

Systematic searches were conducted in MEDLINE, Embase, Scopus, Web of Science, and LILACS from inception through October 2024. The search strategy was structured around three core conceptual groups combined with the Boolean operator AND:

1. Cyclophosphamide exposure or related alkylating agents (“cyclophosphamide”, “alkylating drugs”, “Chemotherapy agent”).
2. Reproductive outcome (“primary ovary insufficiency”, “primary ovarian insufficiency”, “primary ovarian failure”, “premature ovary insufficiency”, “premature ovarian insufficiency”, “premature ovarian failure”, “premature menopause”, “amenorrhea”, “ovarian damage”, “female hypergonadotropic hypogonadism”).
3. Autoimmune disease (“systemic lupus erythematosus”, “lupus”, and “autoimmune disease”).

Controlled vocabulary and free-text keywords were adapted to each database. No search filters were applied, except in Embase, where a research article filter was used. Reference lists of included studies were also hand-searched for additional relevant publications. The full database-specific search strategies, search dates, and number of retrieved records are provided in the **Supplementary Material** (**Supplementary Table 1**).

### 2.3 Study selection and data extraction

The study selection process was carried out in a structured manner to ensure methodological rigor. First, all records retrieved from the different databases were consolidated, and duplicates were carefully removed using the Rayyan platform. The remaining references were then screened at the title and abstract level by two independent reviewers [P.C.G.P., R.C.H.], who applied the predefined eligibility criteria. Articles deemed potentially relevant were subsequently examined in full text to confirm their inclusion. Any discrepancies that arose during the selection process were discussed between the reviewers, and unresolved disagreements were adjudicated by a third reviewer [J.L.E.D.C.].

At full-text review, studies published in English or Spanish were eligible for inclusion. Once the final set of studies was established, data extraction was performed using a standardized form specifically designed for this review. The form included detailed information on study characteristics such as author and year of publication, country, and study design, as well as the period during which the study was conducted (see **Supplementary Tables**). Clinical and methodological variables were also collected, including sample size, number of women with premature ovarian insufficiency, exposure to cyclophosphamide, mean age, and, when available, cumulative CYC dose. This structured approach ensured consistency and comparability across the included studies, laying the foundation for a reliable synthesis of the evidence.

### 2.4 Risk of bias assessment

Methodological quality was assessed independently by two reviewers [E.S.E., F.P.P.] using the Joanna Briggs Institute critical appraisal tool for prevalence studies (20). The tool evaluates nine domains: (1) appropriate sampling frame, (2) proper participant recruitment, (3) adequate sample size, (4) complete subject and setting description, (5) sufficient data coverage, (6) valid measurement methods, (7) standardized measurement application, (8) appropriate outcome definition, and (9) appropriate statistical analysis. Each domain was rated as “Yes”, “No”, or “Unclear”. The results of the risk of bias assessment were summarized and visually presented through a risk of bias graph and a radar plot to facilitate interpretation of methodological strengths and weaknesses across studies.

### 2.5 Synthesis methods

The results were quantitatively synthesized through a meta-analysis, where the primary outcome was the pooled prevalence of POI among women with SLE exposed to cyclophosphamide (CYC), restricted to studies that applied strict diagnostic criteria (amenorrhea plus confirmatory hormonal testing). A secondary analysis included all studies reporting POI irrespective of the definition used.

Pooled prevalence estimates were calculated using random-effects meta-analysis with restricted maximum likelihood (REML) estimation and Hartung-Knapp adjustment. The primary analysis used the Freeman-Tukey double arcsine transformation to stabilize variances (21) in the presence of extreme proportions and zero-event studies. Because this approach may have limitations in back-transformation, we additionally performed sensitivity analyses using a logit transformation with inverse-variance weighting and a generalized linear mixed model (GLMM). Between-study heterogeneity was assessed using Cochran’s Q statistic and corresponding p-value (22), together with I^2^ and tau^2^. Prediction intervals were also calculated.

Subgroup analyses were prespecified according to clinically relevant factors: (a) period (pre-2005 vs. post-2005), reflecting the introduction of the Euro-Lupus low- dose CYC protocol; and (b) age at disease onset (juvenile-onset cohorts). Given that cumulative CYC dose is a major determinant of ovarian toxicity, the Euro-Lupus protocol change provided a meaningful basis for temporal stratification. The Euro-Lupus regimen, first described by Houssiau et al. (23), consists of six biweekly pulses of 500 mg intravenous CYC, representing a substantially lower cumulative exposure compared with the traditional NIH protocol (0.5–1 g/m^2^ monthly for six months). Because the Euro-Lupus low-dose CYC regimen was progressively adopted in clinical practice well before formal guideline incorporation in 2008 (24), we use 2005 as the primary temporal cut-point to approximate the clinical transition toward lower cumulative CYC exposure. Publication year was used as a proxy when recruitment periods were not reported.

The most recent international guidelines (including those from the European Alliance of Associations for Rheumatology (EULAR) (13), ACR (25) and Kidney Disease Improving Global Outcomes (KDIGO) (26)) now recommend the Euro-Lupus low-dose regimen as a preferred first-line induction therapy for lupus nephritis, emphasizing its comparable efficacy and markedly reduced risk of gonadotoxicity. As a sensitivity analysis, we repeated the stratification using 2008 to reflect the first formal appearance in international guidelines.

Additionally, meta-regression models were used to explore the effect of cumulative CYC dose and mean age on POI prevalence. Finally, to account for potential reporting bias, we examined funnel plot asymmetry and applied Egger’s regression test for small-study effects. All analyses were performed in R (version 4.3.3) using the “meta” and “metafor” packages.

## 3 Results

### 3.1 Study selection

**Figure 1:**
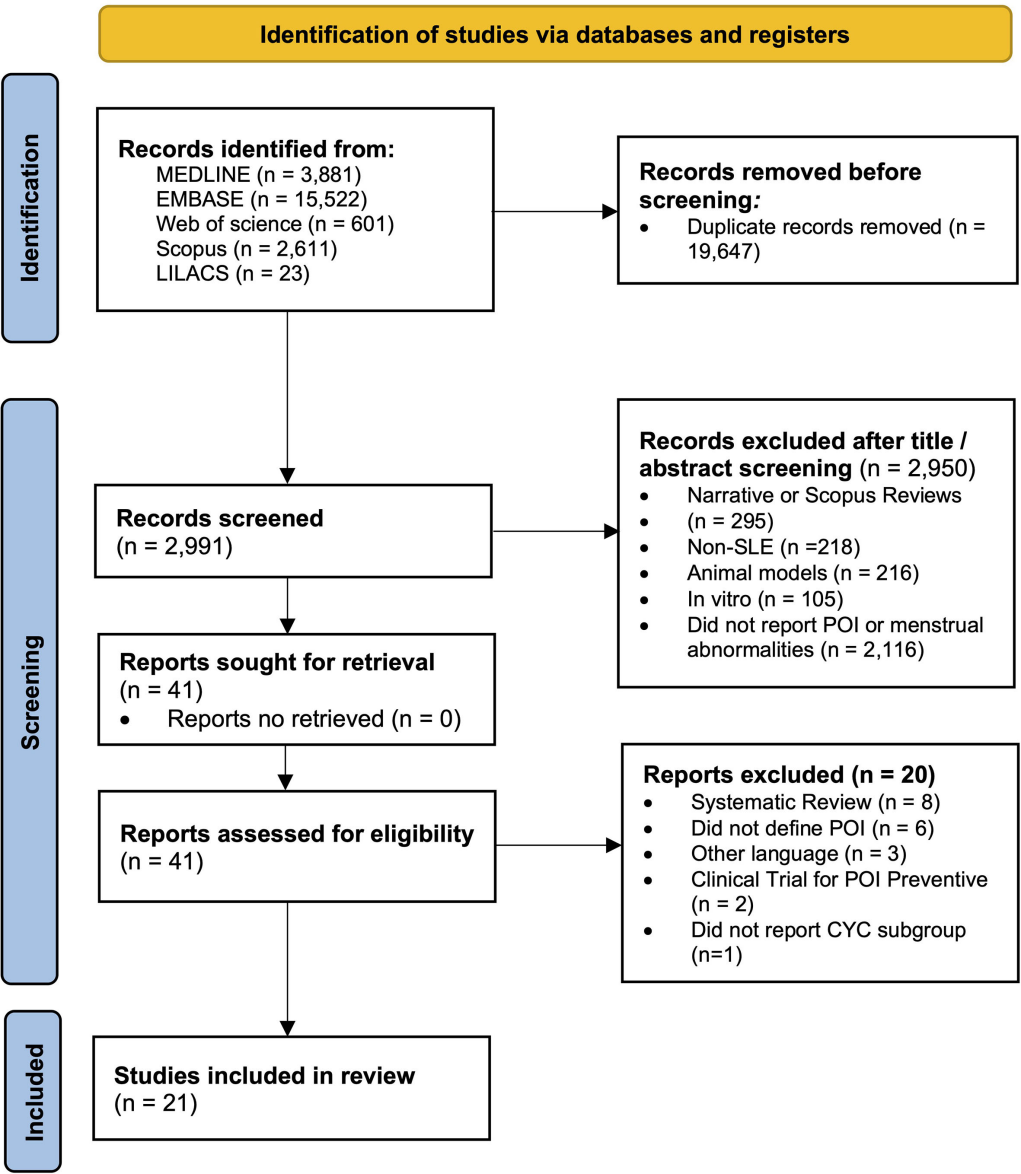
Identification of studies via databases and registers according to PRISMA 2020 guidelines. SLE, Systemic Lupus Erythematosus; POI, Premature Ovarian Insufficiency.

The systematic search yielded 22,638 records, of which 21 studies met eligibility criteria and were included in the systematic review (**Figure 1**). These studies encompassed diverse populations of women with SLE across multiple geographic regions, including North America (10, 27), Europe (7, 28–31), Asia (8, 32–39), and Latin America (2, 11, 40–42). The designs were predominantly observational, comprising both cohort (2, 10, 11, 27–35, 37–39, 41, 42) and cross-sectional studies (7, 8, 36, 40), and collectively spanned more than three decades of research. While all studies explicitly reported premature ovarian insufficiency (POI), the diagnostic approach varied: some relied solely on clinical criteria such as amenorrhea, whereas others applied strict hormonal definitions using FSH, LH, or estradiol testing. To account for this variability, subgroup analyses restricted to studies with strict hormonal criteria were performed and are presented separately. In addition, a supplementary analysis was conducted including all studies that reported POI prevalence in SLE, regardless of cyclophosphamide exposure.

Twelve studies comprising 935 women with SLE treated with CYC contributed to the primary prevalence meta-analysis, while additional studies were incorporated into complementary and subgroup analyses according to treatment exposure and diagnostic definition. Study characteristics are summarized in **Table 1**.

**Table 1:** Overview of all studies included with correct criteria of POI.

Author (Year)	Design	Country	Age (years)	CYC cumulative dose (gr)	Total patients	Events (POI)	Frecuency (%)	Inclusion criteria	POI definition
*McDermott, et al. (1996) (* 29 *)*	Retrospective cohort	United Kingdom	36.1 (mean) (range 17-49 years)	16.8 (median) (IQR 3-62.5)	27	11	41	Treatment with CYC or azathioprine	Premature menopause (< 40 years) with elevated FSH and low estradiol
*Mok, et al. (1998) (* 28 *)*	Retrospective cohort	China	Mean not reported	28.3 (mean) No SD report	70	18	25.7	Women < 45 years with other cause of amenorrhea, treated with CYC or other immunosupresive therapy	Sustained amenorrhea with elevated FSH and LH and low estradiol
*Mok, et al. (1999) (* 33 *)*	Retrospective cohort	China	37.9 (mean) (SD ±1.8)	20.4 (mean) (SD ± 2.2)	54	14	25.9	Women < 45 years, oral CYC for severe manifestations, without uremia and hormonal treatment	Persistent amenorrhea with FSH ≥ 30 U/L, LH ≥ 16 U/L, and estradiol < 30 pmol/L
*Medeiros, et al. (2001) (* 2 *)*	Retrospective cohort	Brazil	35.8 (mean) (SD ±5.4)	18.9 (mean) (SD ± 13.1)	26	9	34.6	Women between 16-45 years, without other causes of amenorrhea, and without chronic renal insufficiency	Amenorrhea > 12 months (< 40 years) with FSH > 42 U/L, LH > 11 U/L, estradiol <14 pg/mL
*Mok, et al. (2006) (* 34 *)*	Retrospective cohort	China	Mean not reported	100 mg/kg (No detail CD)	188	38	20.2	Lupus nephritis IV, treated with oral or IV CYC and prednisolone	Sustained amenorrhea and postmenopausal FSH and estradiol levels
*Silva, et al. (2007) (* 41 *)*	Retrospective cohort	Brazil	No POI	No POI	59	0	0	Women ≥ 10 years, post-menarche, Juvenile-onset SLE, without others causes of amenorrhea	Sustained amenorrhea with FSH ≥ 40 U/L
*Appenzeller, et al. (2008) (* 42 *)*	Retrospective cohort	Brazil	32 (mean) (range 29-39)	16.8 (mean) (range 14-20)	107	10	9.3	Women < 40 years, Treatment with CYC ≥ 2 years before analysis, without other causes of amenorrhea	Sustained amenorrhea with confirmed hormonal testing (FSH, LH, estradiol)
*Mayorga, et al. (2015) (* 11 *)*	Cross-sectional	Mexico	32.7 (mean) (SD ±6.2)	33.2 (mean) (SD ± 49.7)	287	48	16.7	Women < 60 years, without other causes of amenorrhea	Amenorrhea ≥ 12 months before age 40, with FSH elevated
*Akawatcharangura, et al. (2016) (* 8 *)*	Cross-sectional	Thailand	35.8 (mean) (SD ±4.0)	34.9 (mean) (SD ±33.1)	64	10	15.6	≥ 6 months of immunosuppressive therapy, without other causes of amenorrhea	Sustained amenorrhea ≥ 6 months, FSH ≥ 40 U/L, estradiol <30 pg/mL
*Brunner, et al. (2016) (* 27 *)*	Ambispective cohort	USA	No POI	No POI	13	0	0	Childhood-onset SLE, excluded ≥ 19 years, without pregangncy	Secondary amenorrhea ≥ 6 months before age 40, FSH ≥ 40 U/L
*Sharma, et al. (2020) (* 39 *)*	Prospective cohort	India	No POI	No POI	25	0	0	Women between 18-40 years, severe disease, treated with CYC or Mycophenolate, without other causes of amenorrhea	Sustained amenorrhea ≥ 12 months before age 40 and menopausal hormone profile
*Orefice, et al. (2020) (* 31 *)*	Retrospective cohort	Italy	37.7 (mean) (SD ±5.9)	Mean not reported	15	5	33.3	Women < 40 years, treated with CYC	Amenorrhea ≥ 12 months plus FSH ≥ 40 mU/mL before age 40

*All patients have SLE diagnosed by ACR criteria, and other causes of amenorrhea refers to surgical, pregnancy or radiotherapy. SD, Standard deviations; IQR, Interquartile range; POI, Premature ovarian insufficiency; CYC, Cyclophosphamide; FSH, Follicle stimulating hormone; LH, Luteinizing hormone; SLE, Systemic lupus erythematosus; USA, United States of America; CD, Cumulative dose.

### 3.2 Prevalence of POI in patients with SLE

**Figure 2:**
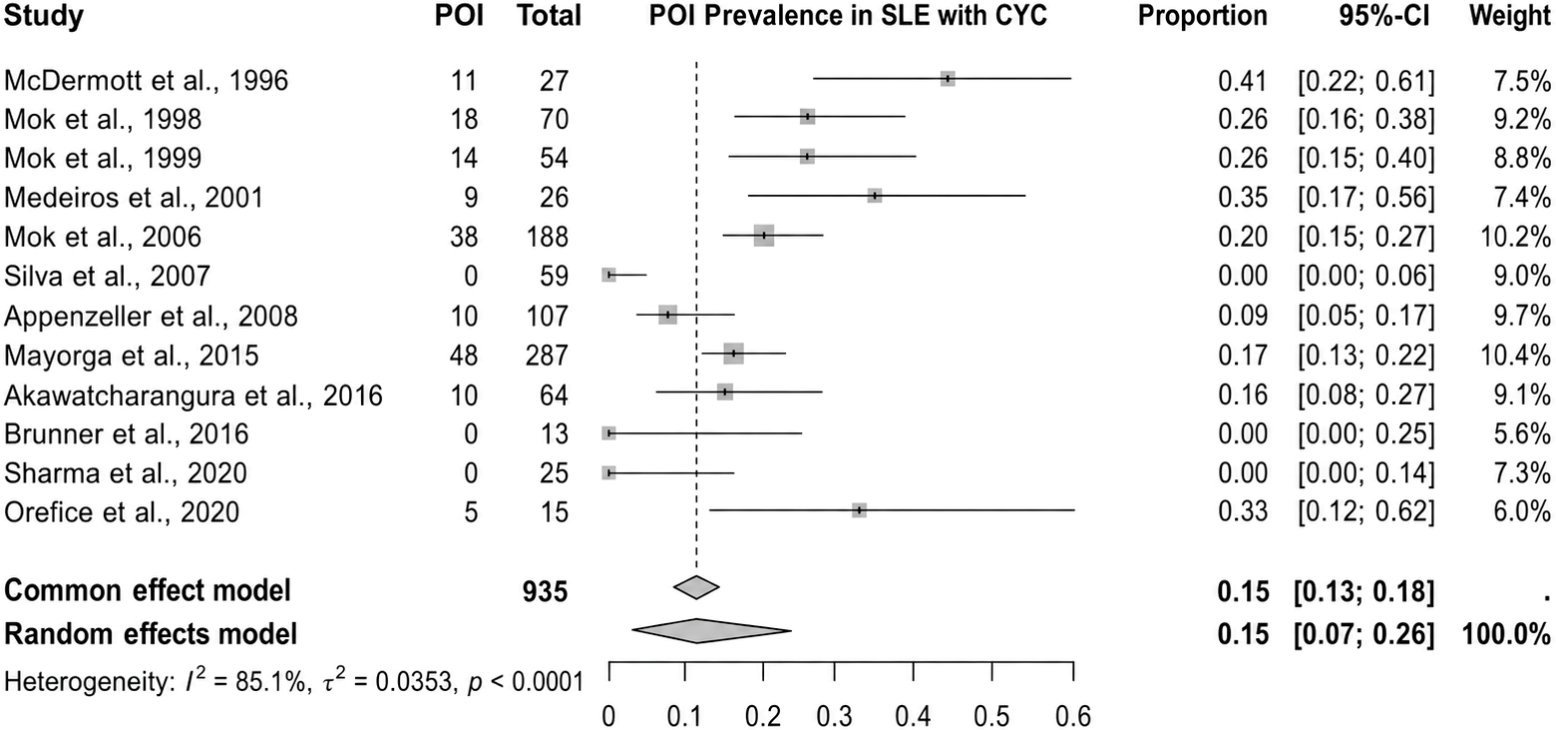
Forest plot of the POI among women with SLE treated with CYC, restricted to studies applying strict diagnostic criteria (amenorrhea with confirmatory hormonal testing).

In the primary meta-analysis including 12 CYC-exposed cohorts (935 women), the pooled prevalence of POI was 15.2% (95% CI 6.5-26.4) under the Freeman-Tukey random-effects model, with substantial between-study heterogeneity (Cochran’s Q = 73.98, p < 0.0001; I2 = 85.1%; tau2 = 0.0353) (**Figure 2**). Sensitivity analyses yielded broadly similar pooled estimates: 20.0% (95% CI 12.7-30.1), using logit transformation with inverse-variance weighting, and 14.5% (95% CI 7.1-27.2) using a generalized linear mixed model. Thus, the overall interpretation of a clinically relevant prevalence of POI among CYC-exposed women with SLE remained unchanged across analytic approaches (**Supplementary Table 2**).

These findings indicate that even when uniform diagnostic definitions are applied, a considerable proportion of women with SLE treated with CYC experience POI. The persistence of substantial heterogeneity suggests that additional factors—such as cumulative CYC dose, age at exposure, disease severity, and variations in treatment protocols—likely contribute to the variability observed across studies.

**Figure 3:**
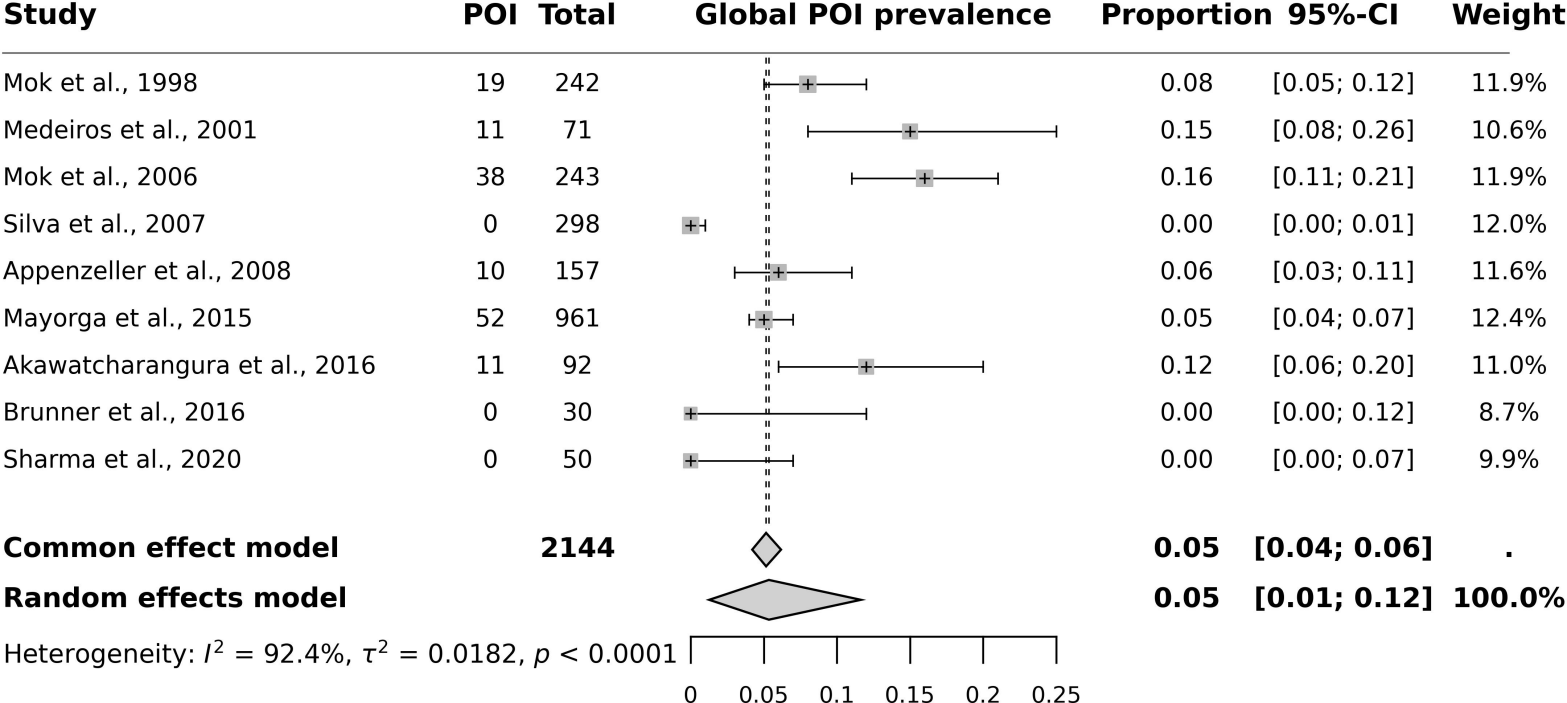
Forest plot illustrating overall prevalence of POI in unselected SLE cohorts, including both CYC- exposed and non-exposed patients, highlighting the higher prevalence in CYC-treated populations.

To further explore the specific impact of CYC exposure, we conducted a complementary analysis that included studies reporting POI prevalence among women with SLE irrespective of treatment status (**Supplementary Table 3**). In this broader population, pooled prevalence was lower (**Figure 3**), which is consistent with an important contribution of CYC exposure but should be interpreted cautiously because this is an indirect comparison across different study sets.

### 3.3 Evaluating the heterogeneity and subgroup effects

Having established that the prevalence of POI is higher among women with SLE exposed to CYC compared with the overall SLE population, we next examined whether treatment modifications over time influenced this risk. The Euro-Lupus protocol, introduced in 2002 and progressively adopted in clinical practice before appearing in EULAR guidance in 2008 (24), provided a lower-dose alternative to conventional regimens. Because not all studies reported the precise recruitment period, the year of publication was used as a proxy to stratify cohorts into pre-2005 and post-2005 groups.

**Figure 4:**
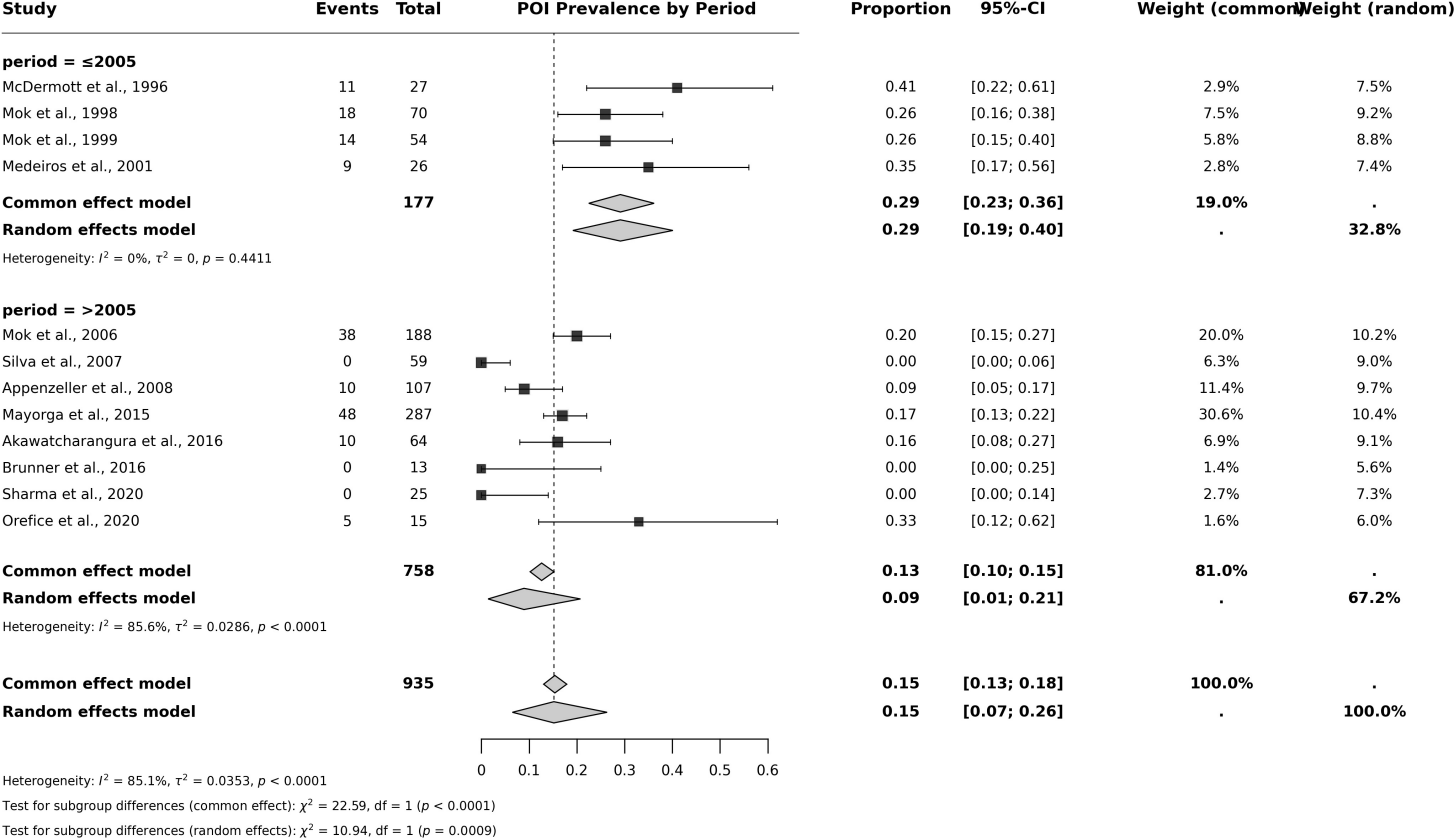
Forest plot of the prevalence of POI in women with SLE treated with CYC, stratified by study period (pre-2005 vs. post-2005). Publication year was used as a proxy when recruitment timeframe was not reported. The reduction in prevalence observed after 2005 coincides with the adoption of the Euro-Lupus low-dose CYC regimens; see **Supplementary Material** for the 2008 and 2019 sensitivity cut-point.

A key finding of this study was the marked temporal decline in POI prevalence, from 29% (95% CI 19-40%) in studies published before 2005 to 9% (95% CI 1-21%) thereafter (**Figure 4**). Heterogeneity was minimal among pre-2005 studies, suggesting that treatment protocol and cumulative CYC dose may be major determinants of gonadal failure risk.

To further assess the temporal effect, sensitivity analyses were conducted using alternative cut- points. When using 2008, the year when the Euro-Lupus regimen was first cited in EULAR guidance (24), the pooled prevalence remained lower (10%, 95% CI 1-24.7%), confirming a sustained downward trend. In contrast, stratification by 2019 (the year of formal guideline endorsement by EULAR) included only two post-2019 studies and yielded wide confidence intervals (10.3%, 95% CI 0-100%) with no significant subgroup difference (p = 0.80).

These findings indicate that the decline in POI prevalence preceded formal guideline adoption, reflecting earlier clinical uptake of low-dose regimens. Full outputs are provided in **Supplementary Figures 1**, **2**.

**Figure 5:**
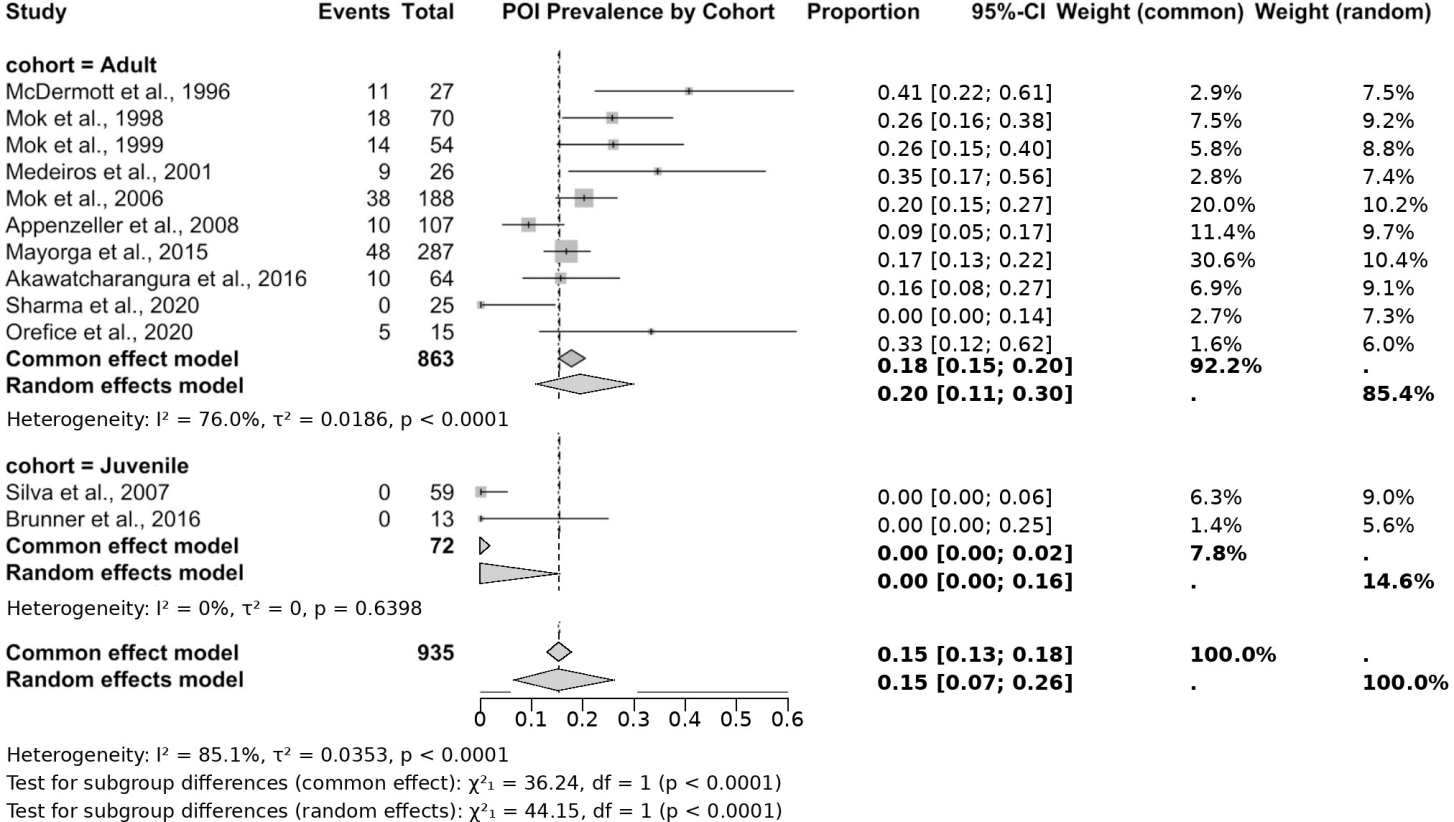
Forest plot of POI prevalence in women with SLE treated with CYC stratified by age at disease onset (adult-onset vs juvenile-onset cohorts), demonstrating the markedly lower prevalence in younger women, consistent with the protective effect of higher baseline ovarian reserve.

Given the strong evidence from the literature suggesting that younger age is protective against CYC- induced ovarian toxicity, we next explored the prevalence of POI in juvenile-onset SLE. Notably, two of the three studies (27, 39, 41) that reported no cases of POI were conducted in juvenile cohorts, reinforcing the hypothesis that younger age may mitigate the risk of gonadal damage. Based on these observations, we performed a subgroup analysis restricted to juvenile-onset SLE populations. As shown in **Figure 5**, the pooled prevalence of POI in this subgroup was substantially lower than in adult cohorts, consistent with the protective effect of higher ovarian reserve at younger ages.

Given the variability in POI definitions across the included studies, we performed an additional subgroup analysis stratifying studies according to diagnostic criteria. One group comprised studies applying strict hormonal definitions (amenorrhea plus elevated gonadotropins and/or low estradiol), whereas the other included studies using broader, clinically oriented definitions, most often based on sustained amenorrhea, which more closely resembles the gonadal damage definition incorporated in the SLICC Damage Index (**Supplementary Table 4**).

The pooled prevalence among studies using strict hormonal criteria was 15.2% (95% CI: 6.5-26.4%), whereas studies using broader clinical definitions yielded a somewhat higher pooled prevalence of 19% (95% CI: 10-31%). These findings suggest that definitional heterogeneity may contribute to variability in prevalence estimates and should be considered when comparing rheumatologic and endocrinologic literature. However, because the strength of the between-subgroup difference was attenuated in logit-based sensitivity analyses, this subgroup finding should be interpreted cautiously (**Supplementary Figure 3**).

To further explore potential sources of heterogeneity, we conducted meta-regression analyses examining cumulative CYC dose and mean age at exposure. Cumulative dose was not significantly associated with POI prevalence (β≈ -0.0002; 95% CI: -0.075 to 0.075; p = 0.996), and residual heterogeneity remained high (I^2^ = 81%), suggesting that incomplete or inconsistent reporting of cumulative exposure across studies limited the ability to detect dose-response effects (**Supplementary Figure 4**).

**Figure 6:**
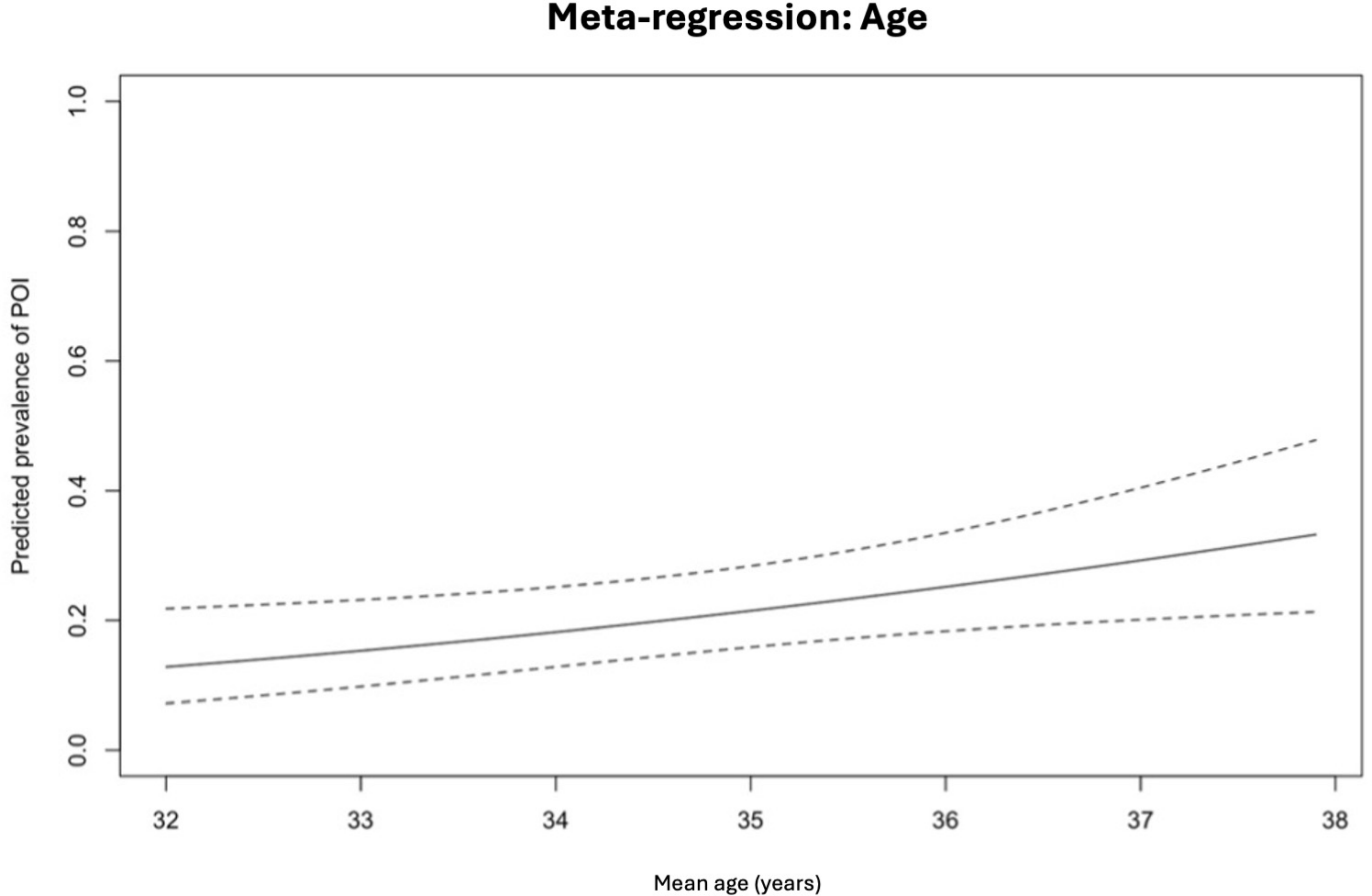
Meta-regression illustrating the association between age and the prevalence of POI in women with SLE treated with CYC.

In contrast, age at exposure emerged as a significant predictor. When restricted to studies with consistent reporting, each additional year of age increased the prevalence of POI by approximately 20% (β = 0.21; 95% CI: 0.04-0.38; p = 0.017), accounting for ∼60% of between-study heterogeneity (residual I^2^ = 54%) (**Figure 6**). This finding highlights that older age at CYC exposure is an independent risk factor for gonadal failure, consistent with the biological decline in ovarian reserve with advancing age.

### 3.4 Risk of bias

**Figure 7:**
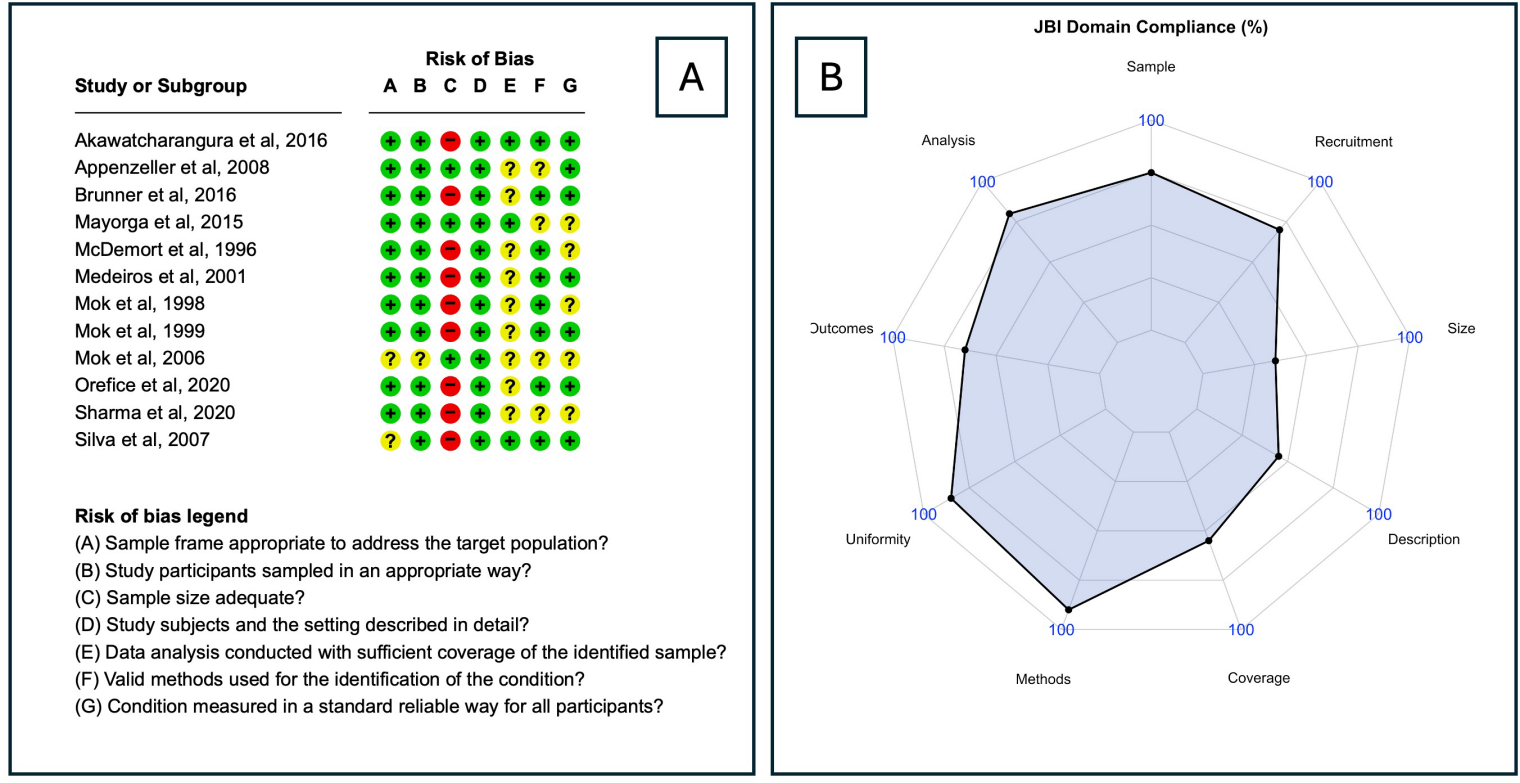
Risk of bias assessment of included studies using the JBI checklist for prevalence studies. **(A)** Domain- specific ratings for each study (green = low risk, yellow = unclear, red = high risk). **(B)** Radar plot summarizing the proportion of studies meeting each JBI criterion.

Methodological quality was assessed using the Joanna Briggs Institute checklist for prevalence studies. As illustrated in **Figure 7**, compliance was highest for domains related to study description, recruitment, analysis, and outcome measurement (>80%). In contrast, only 45% of studies were considered to have an adequate sample size, and population coverage was limited in approximately half of the included cohorts. The risk of bias plot revealed considerable variability across individual studies, particularly in domains related to sampling frame and representativeness, while the radar plot highlighted consistent strengths in outcome measurement but recurrent weaknesses in external validity. Taken together, these findings indicate that although most studies were methodologically sound in their internal conduct, concerns remain regarding sample adequacy and generalizability.

### 3.5 Publication bias

**Figure 8:**
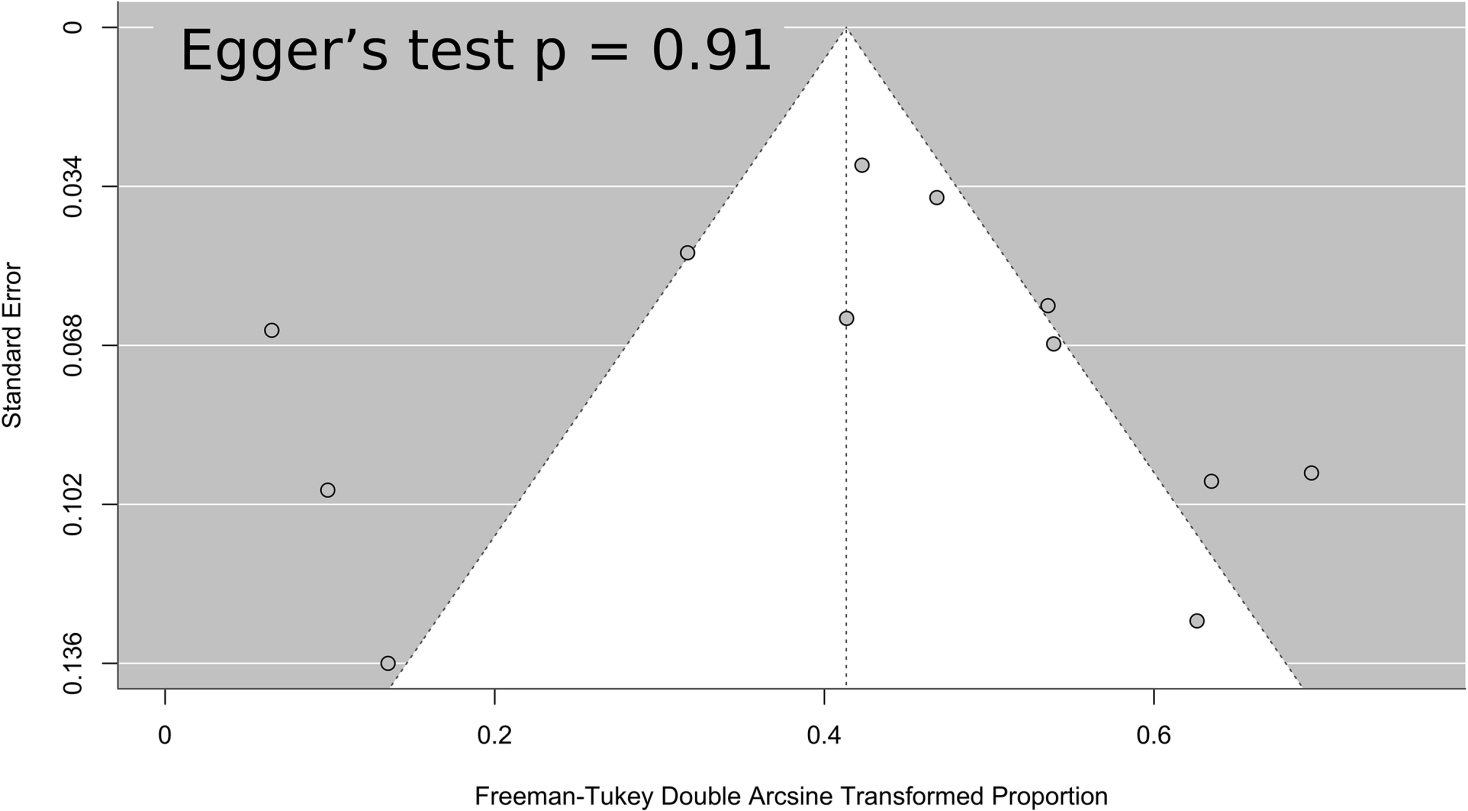
Funnel plot of studies reporting the prevalence of POI in women with SLE treated with CYC, showing no evidence of asymmetry or publication bias.

Potential publication bias was assessed through visual inspection of the funnel plot and formal statistical testing. The funnel plot (**Figure 8**) did not reveal evidence of asymmetry, and Egger’s regression test for small-study effects was not significant (τ = -0.11, df = 10, p = 0.91). These findings suggest a low likelihood of publication bias influencing the pooled prevalence estimates.

## 4 Discussion

This systematic review and meta-analysis demonstrate that POI in women with SLE is not an inevitable consequence of therapy, but rather a long-term damage outcome strongly shaped by treatment era and age at exposure. Across more than three decades of published evidence, we observed a marked decline in POI prevalence following the early adoption of lower-dose CYC regimens, with pooled estimates decreasing from nearly one-third of exposed patients in studies published before 2005 to less than 10% thereafter. Importantly, age at exposure emerged as an independent determinant of risk, explaining a substantial proportion of between-study heterogeneity, whereas cumulative CYC alone did not consistently predict ovarian failure. Taken together, these findings suggest that the reproductive risk profile of women with SLE has evolved in parallel with changes in therapeutic strategies, supporting the view of POI as a modifiable component of cumulative damage rather than a fixed adverse effect of disease or treatment.

Our findings are broadly consistent with the most recent systematic review and meta-analysis by Giambalvo et al. (2022), which included 46 studies and 4,704 women with SLE. That review identified CYC exposure and cumulative dose as the strongest predictors of ovarian failure. Importantly, it also emphasized the wide variability in POI definitions, ranging from purely clinical amenorrhea to endocrine-based criteria using FSH, LH, and estradiol (43). Our analysis corroborates this heterogeneity and underscores its direct impact on pooled prevalence estimates. Compared with prior meta- analyses, our study contributes novel subgroup analyses (pre- vs. post-2005, juvenile- vs. adult-onset SLE, strict vs. broad POI definitions), illustrating how treatment evolution, patient age, and definitional choices influence prevalence estimates.

Ethnicity and geographic context may also influence the risk of POI in women with SLE. Some cohorts have suggested higher vulnerability in certain populations, potentially reflecting differences in disease severity, treatment access, cumulative exposure, socioeconomic factors, and genetic susceptibility. However, ethnicity was inconsistently reported across the included studies, which precluded a reliable pooled subgroup or meta-regression analysis. Accordingly, we interpret ethnicity as an important but insufficiently characterized source of heterogeneity that warrants dedicated prospective evaluation in more globally representative cohorts.

Clinically, these findings reaffirm that ovarian toxicity remains a critical concern in the management of women with SLE requiring CYC. The decline in POI prevalence observed after 2005 supports the protective role of low-dose regimens and highlights the need for individualized treatment selection.

The implications of these findings also extend to ovarian-protective strategies during CYC treatment. GnRHa have been used as co-therapy to reduce ovarian exposure to cytotoxic damage, and their role is increasingly recognized in lupus care. Although our meta-analysis was not designed to quantify the independent effect of GnRHa, the temporal decline in POI prevalence observed in more recent cohorts is compatible with a broader shift toward gonadotoxicity-sparing practice, which may include lower cumulative CYC exposure, improved patient selection, and more frequent use of fertility-preservation options, particularly in women at older reproductive ages or those expected to receive repeated CYC exposure.

Age at exposure emerged as an independent determinant of risk, aligning with the biological decline of ovarian reserve. These observations emphasize the importance of fertility preservation strategies— including GnRHa and cryopreservation—particularly in women at higher risk.

Moreover, POI in SLE is not merely a reproductive outcome but a systemic health issue. Unlike other causes of amenorrhea, true POI entails persistent hypoestrogenism, which accelerates bone loss, increases cardiovascular morbidity, and worsens metabolic outcomes (16, 44–46). Given that women with SLE are already at elevated risk of osteoporosis, thrombosis, and premature cardiovascular disease, the additive burden of POI carries profound long-term consequences. Accurate diagnosis is therefore essential for reproductive counseling, fertility preservation, and preventive strategies addressing bone and cardiovascular health (1).

From a methodological perspective, definitional heterogeneity of POI remains a key source of variability. Early rheumatology studies (e.g., Langevitz 1992 (32), Mok 1998/1999 (28, 33)) defined “premature ovarian failure” as sustained amenorrhea before age 40, without hormonal confirmation. The SLICC Damage Index similarly included gonadal failure as ≥6 months of non-surgical amenorrhea or menopause before age 40 (47). In the 2000s, several cohorts (e.g., Medeiros 2001 (2), Mok 2006 (34), Appenzeller 2008 (42)) incorporated hormonal testing with FSH, LH, and estradiol, while still relying on amenorrhea as the primary marker. By contrast, the ESHRE guidelines (2016 and update 2025 (3, 48)) defined POI as ≥4 months of oligo/amenorrhea with elevated FSH levels (>25 U/L) on two occasions before age 40 and acknowledged the possibility of intermittent ovarian function. The most recent multisociety guideline jointly developed by ESHRE, ASRM, Centre for Research Excellence in Women’s Health in Reproductive Life (CRE-WHiRL) and the International Menopause Society (IMS) converges on these diagnostic criteria and incorporate biomarkers such as AMH and antral follicle count (3). This historical divergence explains why prevalence estimates in SLE vary depending on whether rheumatologic “damage” definitions or endocrinologic diagnostic criteria were applied.

The additional sensitivity analyses strengthen the robustness of the main findings. In the primary 12-study analysis, the pooled prevalence obtained with Freeman-Tukey transformation (15.2%) was very similar to that obtained with GLMM (14.5%), whereas the logit inverse-variance model yielded a somewhat higher estimate (20.0%). This variation is likely related to sparse-data behavior and continuity correction in studies with zero events. Importantly, the overall interpretation did not materially change across methods. Nevertheless, the variability across analytic methods underscores the importance of interpreting pooled prevalence estimates cautiously in the presence of small studies and zero-event cohorts.

The temporal cut-point used in this meta-analysis (pre-2005 vs post-2005) was based on the period when the Euro-Lupus low-dose CYC regimen began to be adopted in clinical practice following its publication in 2002 (23). Although the 2008 EULAR guideline (24) cited the Euro-Lupus trial as supporting evidence, the low-dose regimen was not yet incorporated into its formal treatment recommendations. The first official inclusion of this approach in international guidelines occurred with the 2019 EULAR lupus nephritis guideline (49). Therefore, our 2005 cut-point should be interpreted as an empirical marker of the early clinical transition toward reduced cumulative CYC exposure rather than the precise year of guideline endorsement. This approximation was necessary because many primary studies did not report recruitment periods in sufficient detail. Although the 2019 EULAR guideline formally endorsed the Euro-Lupus low-dose regimen, most primary studies published after that date included patients treated in earlier years. Consequently, the 2019 cut point served only as a normative reference rather than an indicator of real-world protocol change.

Strengths of this review include the comprehensive search strategy, the use of subgroup and sensitivity analyses, and the application of meta-regression to address heterogeneity. Nevertheless, several limitations must be acknowledged. Most included studies were observational, with inherent risks of selection and reporting bias. Heterogeneity remained high in many analyses, even after stratification. Definitional variability of POI limited comparability, and lack of individual patient data prevented more precise modeling of dose–response relationships. In addition, language eligibility was restricted to English and Spanish at full-text selection, which may have introduced language bias. Amenorrhea in SLE occasionally reflect central hypogonadism or autoimmune hypophysitis rather than primary ovarian failure, although this is uncommon. Finally, although publication bias was not detected, small-study effects cannot be completely excluded.

Future research should prioritize harmonization of POI definitions across rheumatology and endocrinology, ideally reporting both SLICC-based gonadal damage and ESHRE-based diagnostic criteria. Prospective studies with standardized assessment of ovarian reserve (AMH, AFC) and long- term reproductive outcomes are urgently needed. Individual patient data meta-analyses would help clarify dose–response thresholds and the interaction between age and cumulative CYC exposure. Finally, investigation into genetic determinants of CYC toxicity (50–52) and protective interventions (e.g., GnRH analogues (15), cryopreservation (53)) represent critical avenues for advancing both personalized treatment and fertility preservation in women with SLE.

## 5 Conclusions

This meta-analysis shows that approximately 15% of women with SLE exposed to CYC present POI, with higher prevalence observed in older patients and in cohorts published before the adoption of low-dose Euro-Lupus regimens. The variability in prevalence across studies reflects both clinical determinants—such as age at exposure and treatment protocols—and longstanding definitional differences between rheumatology and endocrinology. Beyond its reproductive implications, POI is a marker of systemic health burden, adding to the elevated risks of osteoporosis, cardiovascular disease, and metabolic complications in women with SLE. These findings underscore the need for harmonized diagnostic criteria, individualized treatment approaches, structured follow-up, and integration of fertility preservation strategies into routine lupus care.

## Data Availability

The original contributions presented in the study are included in the article/**Supplementary Material**. Further inquiries can be directed to the corresponding author.
