# Supplementary material for: Premature ovarian insufficiency in patients with systemic lupus erythematosus on cyclophosphamide: a systematic review and meta-analysis
Source: Front Endocrinol (Lausanne). 2026 May 28;17:1775060. doi: 10.3389/fendo.2026.1775060 (PMC13253310; doi:10.3389/fendo.2026.1775060)
Supplement: Supplementary file 1 [file SupplementaryFile1.docx]

**Supplementary material to:**

**Premature Ovarian Insufficiency in patients with Systemic Lupus Erythematosus on cyclophosphamide: A Systematic review & meta-analysis.**

Manuel Ramón García-Sáenz^1^, Claudia Ramírez-Rentería^2^, Pattsy Etual Espinosa-Cárdenas^1^, Ernesto Sosa-Eroza^1^, José Luis Eduardo Doval-Caballero^3^, Paulo César Gete Palacios^2^, Fabiola Pazos-Pérez^4^, Mario César Ocampo-Torres^5^, Rocío Catana-Hernández^5^, Miguel Ángel Vázquez-Zaragoza^5^, Adolfo Camargo-Coronel^5^, Juan Rodrigo Gómez-Bernal^6^, Aldo Ferreira-Hermosillo^2^.

^1^Departamento de Endocrinología, Hospital de Especialidades del Centro Médico Nacional Siglo XXI, Instituto Mexicano del Seguro Social, Mexico City, 06720, Mexico.

^2^Unidad de Investigación Médica en Enfermedades Endocrinas, Hospital de Especialidades del Centro Médico Nacional Siglo XXI, Instituto Mexicano del Seguro Social, Mexico City, 06720, Mexico.

^3^Obesity clinic, Instituto Nacional de Ciencias Médicas y Nutrición “Salvador Zubirán”, Mexico City, 14080, Mexico.

^4^Departamento de Nefrología, Hospital de Especialidades del Centro Médico Nacional Siglo XXI, Instituto Mexicano del Seguro Social, Mexico City, 06720, Mexico.

^5^Departamento de Reumatología, Hospital de Especialidades del Centro Médico Nacional Siglo XXI, Instituto Mexicano del Seguro Social, Mexico City, 06720, Mexico.

^6^Unidad de Posgrados, Facultad de Medicina, Universidad Nacional Autónoma de México, Mexico City, 04510, Mexico.

| **Supplementary Table 1.** Summary of information sources, core search concepts, and records retrieved | | | |  |
| --- | --- | --- | --- | --- |
| **Database** | **Coverage** | **Core search concepts** | **Records retrieved** | |
| MEDLINE | Inception to October 2024 | The electronic search strategy was structured around three core conceptual groups combined with the Boolean operator AND:   - Cyclophosphamide OR Alkylating drugs OR Chemotherapy agent - Primary ovary insufficiency OR Primary ovarian insufficiency OR Primary ovarian failure OR Primary ovary failure OR Premature ovarian insufficiency OR Premature ovary insufficiency OR Premature ovarian failure OR Premature ovary failure OR Premature menopause OR Amenorrhea OR Ovarian damage - Systemic lupus erythematosus OR Lupus OR Autoimmune disease   The search was performed using combinations of terms from any of these three conceptual groups. | 3881 | |
| Embase | Inception to October 2024 | Same concepts adapted to Emtree terms. | 15522 | |
| Web of Science | Inception to October 2024 | Same concepts adapted to database-specific keywords. | 601 | |
| Scopus | Inception to October 2024 | Same concepts adapted to database-specific keywords. | 2611 | |
| LILACS | Inception to October 2024 | Same concepts adapted to database-specific subject headings and keywords. | 23 | |

Searches were structured around three conceptual groups combined with AND: cyclophosphamide/alkylating agents, primary ovarian insufficiency-related outcomes, and systemic lupus erythematosus/autoimmune disease terms. Controlled vocabulary and free-text terms were adapted to each database. No search filters were applied, except in Embase, where a research article filter was used.

| **Supplementary Table 2.** Sensitivity analyses of the primary pooled prevalence estimate according to meta-analytic method | | | | | |
| --- | --- | --- | --- | --- | --- |
| **Method** | **Pooled prevalence (95% CI)** | **Prediction interval** | **I^2^** | **Tau^2^** | **Q (p-value)** |
| Freeman-Tukey | 15.2 % (6.5-26.4) | 0.0-56.3 % | 85.1 % | 0.0353 | 73.98 (<0.0001) |
| Logit-IV | 20.0 % (12.7-30.1) | 6.9-45.8 % | 68.2 % | 0.2674 | 34.59 (0.0003) |
| GLMM | 14.5 % (7.1-27.2) | 1.4-66.9 % | 54.0 % | 1.1391 | Wald 23.93 (0.0130) |

This table summarizes the pooled prevalence of primary ovarian insufficiency (POI) in cyclophosphamide-exposed women with systemic lupus erythematosus (SLE) using three analytical approaches: Freeman-Tukey double arcsine transformation, logit-transformed inverse-variance model, and generalized linear mixed model (GLMM). For each method, the pooled prevalence, 95% confidence interval (CI), prediction interval, and heterogeneity measures are shown. Heterogeneity was assessed using Cochran’s Q statistic, I^2^, and tau^2^.

Abbreviations: CI, confidence interval; GLMM, generalized linear mixed model; IV, inverse variance; POI, premature ovarian insufficiency; SLE, systemic lupus erythematosus.

| **Supplementary Table 3.** Overview of all studies that report POI independent of treatment received | | | | | | | | | |
| --- | --- | --- | --- | --- | --- | --- | --- | --- | --- |
| **Author (Year)** | **Design** | **Country** | **Age (years)** | **CYC cumulative dose (gr)** | **Total patients** | **Events (POI)** | **Frecuency (%)** | **Inclusion criteria** | **POI Definition** |
| Mok, et al. (1998)^1^ | Retrospective cohort | China | Mean not reported | 28.3 (mean)  No SD report | 242 | 19 | 7.85 | Women < 45 years with other cause of amenorrhea, treated with CYC or other immunosupresive therapy | Sustained amenorrhea with elevated FSH and LH and low estradiol |
| Medeiros, et al. (2001)^2^ | Retrospective cohort | Brazil | 35.8 (mean)  (SD ±5.4) | 18.9 (mean)  (SD ± 13.1) | 71 | 11 | 15.49 | Women between 16-45 years, without other causes of amenorrhea, and without chronic renal insufficiency | Amenorrhea > 12 months (< 40 years) with FSH > 42 U/L, LH > 11 U/L, estradiol <14 pg/mL |
| Mok, et al. (2006)^3^ | Retrospective cohort | China | Mean not reported | 100 mg/kg  (No detail CD) | 243 | 38 | 15.64 | Lupus nephritis IV, treated with oral or IV CYC and prednisolone | Sustained amenorrhea and postmenopausal FSH and estradiol levels |
| Silva, et al. (2007)^4^ | Retrospective cohort | Brazil | No POI | No POI | 298 | 0 | 0 | Women ≥ 10 years, post-menarche, Juvenile-onset SLE, without others causes of amenorrhea | Sustained amenorrhea sith FSH ≥ 40 U/L |
| Appenzeller, et al. (2008)^5^ | Retrospective cohort | Brazil | 32 (mean)  (range 29-39) | 16.8 (mean)  (range 14-20) | 157 | 10 | 6.37 | Women < 40 years, Treatment with CYC ≥ 2 years before analysis, without other causes of amenorrhea | Sustained amenorrhea with confirmed hormonal testing (FSH, LH, estradiol) |
| Mayorga, et al. (2015)^6^ | Cross-sectional | Mexico | 32.7 (mean)  (SD ±6.2) | 33.2 (mean)  (SD ± 49.7) | 961 | 52 | 5.41 | Women < 60 years, without other causes of amenorrhea | Amenorrhea ≥ 12 months before age 40, with FSH elevated |
| Akawatcharangura, et al. (2016)^7^ | Cross-sectional | Thailand | 35.8 (mean)  (SD ±4.0) | 34.9 (mean)  (SD ±33.1) | 92 | 11 | 11.96 | ≥ 6 months of immunosupresor therapy, without other causes of amenorrhea | Sustained amenorrhea ≥ 6 months, FSH ≥ 40 U/L, estradiol <30 pg/mL |
| Brunner, et al. (2016)^8^ | Ambispective cohort | USA | No POI | No POI | 30 | 0 | 0 | Childhood-onset SLE, excluded ≥ 19 years, without pregangncy | Secondary amenorrhea ≥ 6 months before age 40, FSH ≥ 40 U/L |
| Sharma, et al. (2020)^9^ | Prospective cohort | India | No POI | No POI | 50 | 0 | 0 | Women between 18-40 years, severe disease, treated with CYC or Micophenolate, without other causes of amenorrhea | Sustained amenorrhea ≥ 12 months before age 40 and menopausal hormone profile |

*All patients have SLE diagnostic by ACR criteria, and other causes of amenorrhea means to surgical, pregnancy or radiotherapy. SD = Standard deviations, IQR = Interquartile range, POI = Premature ovarian insufficiency, CYC = Cyclophosphamide, FSH = Follicle stimulating hormone, LH = Luteinizing hormone, SLE = Systemic lupus erythematosus, USA = United States of America, CD = Cumulative dose.

| **Supplementary Table 4.** Overview of all studies that POI report but the definition not include hormonal profile | | | | | | | | | |
| --- | --- | --- | --- | --- | --- | --- | --- | --- | --- |
| **Author (Year)** | **Design** | **Country** | **Age (years)** | **CYC cumulative dose (gr)** | **Total patients** | **Events (POI)** | **Frecuency (%)** | **Inclusion criteria** | **POI Definition** |
| Langervits, et al. (1992)^10^ | Retrospective case series | Israel | 28 | 11 | 17 | 1 | 5.8 | Lupus nephritis:  nephrotic syndrome, glucocorticoid or azatioprine not response | Premature menopause in women of 28 years and sustained amenorrhea |
| Park, et al. (2004) | Retrospective cohort | Korea | 35.1 (mean)  (SD ±5.0) | 9.4 (mean)  (SD ± 2.2) | 67 | 10 | 14.9 | Lupus nephritis III or IV, other causes of amenorrhea, uremia | Permanent amenorrhea |
| Velásquez Méndez, et al. (2006)^11^ | Cross-sectional | Colombia | 35.2 (mean)  No SD report | 7.36 (mean)  No SD report | 56 | 9 | 16.1 | Women < 40 años, without other causes of amenorrhea, without chronic renal failure, and without hormonal therapy | Sustained amenorrhea |
| Singh, et al. (2007)^12^ | Cross-sectional | India | 26.12 (mean)  (SD ±8.6) | 9.1 (mean)  (SD ±2.9) | 35 | 11 | 31.4 | Treatment with CYC (≥ 6 pulses) and 1 year follow-up, without other causes of amenorrhea | Amenorrhea ≥12 months before age 45 years, with menopausal hormones |
| González, et al. (2008)^13^ | Prospective cohort | USA | 28.8 (mean)  (SD ±6.1) | Mean not reported | 76 | 25 | 32.9 | Women between 16-40 years, disease duration ≤ 5 years at baseline, without other causes of amenorrhea | Menopause before age 40, regardless of cause |
| Bozzolo, et al. (2013)^14^ | Retrospective cohort | Italy | 32.7 (mean)  (SD ±4.7) | 20.8 (mean)  (SD ± 6.4) | 29 | 6 | 20.7 | Lupus nephritis (class III, IV or V), adequate follow-up | Sustained amenorrhea, no strict hormonal criteria detailed in the text |
| Alarfaj, et al. (2014)^15^ | Retrospective cohort | Saudite Arabian | 32.4 (mean)  (SD ±7.9) | 8.3 (mean)  (SD ± 2.3) | 99 | 6 | 6.1 | Premenopausal women followed over 26 years, without other causes of amenorrhea | Sustained amenorrhea > 12 months before age 40 |
| Kothari, et al. (2016)^16^ | Ambispective cohort | India | ≥ 30 | ≥ 7 | 27 | 3 | 11.1 | Women ≥ 13 years, without other connective tissue diseases or ginecological disorder | Irreversible amenorrhea, menopause premature |
| Ceccareli, et al. (2020)^17^ | Cross-sectional | Italy | Mean not reported | Mean not reported | 17 | 9 | 52.9 | Patients with follow-up in Sapienza Lupus Cohort | Amenorrhea ≥ 12 months before age 40 |

*All patients have SLE diagnostic by ACR criteria, and other causes of amenorrhea means to surgical, pregnancy or radiotherapy. SD = Standard deviations, IQR = Interquartile range, POI = Premature ovarian insufficiency, CYC = Cyclophosphamide, FSH = Follicle stimulating hormone, LH = Luteinizing hormone, SLE = Systemic lupus erythematosus, USA = United States of America, CD = Cumulative dose.

Referencias

1. Mok CC, Lau CS, Wong RW. Risk factors for ovarian failure in patients with systemic lupus erythematosus receiving cyclophosphamide therapy. *Arthritis Rheum*. May 1998;41(5):831-7. doi:10.1002/1529-0131(199805)41:5<831::AID-ART9>3.0.CO;2-1

2. Medeiros MM, Silveira VA, Menezes AP, Carvalho RC. Risk factors for ovarian failure in patients with systemic lupus erythematosus. *Braz J Med Biol Res*. Dec 2001;34(12):1561-8. doi:10.1590/s0100-879x2001001200008

3. Mok CC, Ying KY, Ng WL, et al. Long-term outcome of diffuse proliferative lupus glomerulonephritis treated with cyclophosphamide. *Am J Med*. Apr 2006;119(4):355 e25-33. doi:10.1016/j.amjmed.2005.08.045

4. Silva CA, Hilario MO, Febronio MV, et al. Risk factors for amenorrhea in juvenile systemic lupus erythematosus (JSLE): a Brazilian multicentre cohort study. *Lupus*. 2007;16(7):531-6. doi:10.1177/0961203307079300

5. Appenzeller S, Blatyta PF, Costallat LT. Ovarian failure in SLE patients using pulse cyclophosphamide: comparison of different regimes. *Rheumatol Int*. Apr 2008;28(6):567-71. doi:10.1007/s00296-007-0478-3

6. Mayorga J, Alpizar-Rodriguez D, Prieto-Padilla J, Romero-Diaz J, Cravioto MC. Prevalence of premature ovarian failure in patients with systemic lupus erythematosus. *Lupus*. Jun 2016;25(7):675-83. doi:10.1177/0961203315622824

7. Akawatcharangura P, Taechakraichana N, Osiri M. Prevalence of premature ovarian failure in systemic lupus erythematosus patients treated with immunosuppressive agents in Thailand. *Lupus*. Apr 2016;25(4):436-44. doi:10.1177/0961203315617539

8. Brunner HI, Bishnoi A, Barron AC, et al. Disease outcomes and ovarian function of childhood-onset systemic lupus erythematosus. *Lupus*. 2006;15(4):198-206. doi:10.1191/0961203306lu2291oa

9. Sharma SK, Jain S, Bahl P, et al. Ovarian dysfunction with moderate-dose intravenous cyclophosphamide (modified NIH regimen) and mycophenolate mofetil in young adults with severe lupus: a prospective cohort study. *Arthritis Res Ther*. Aug 14 2020;22(1):189. doi:10.1186/s13075-020-02292-y

10. Langevitz P, Klein L, Pras M, Many A. The effect of cyclophosphamide pulses on fertility in patients with lupus nephritis. *Am J Reprod Immunol*. Oct-Dec 1992;28(3-4):157-8. doi:10.1111/j.1600-0897.1992.tb00780.x

11. Velásquez Méndez MP RRF, Vásquez Duque GM, Ramírez Gómez LA. Prevalencia de falla ovárica y factores de riesgo en pacientes con Lupus Eritematoso Sistémico tratadas con ciclofosfamida intravenosa. *Revista Colombiana de Reumatología*. 2006;13(3):9.

12. Singh G, Saxena N, Aggarwal A, Misra R. Cytochrome P450 polymorphism as a predictor of ovarian toxicity to pulse cyclophosphamide in systemic lupus erythematosus. *J Rheumatol*. Apr 2007;34(4):731-3.

13. Gonzalez LA, McGwin G, Jr., Duran S, et al. Predictors of premature gonadal failure in patients with systemic lupus erythematosus. Results from LUMINA, a multiethnic US cohort (LUMINA LVIII). *Ann Rheum Dis*. Aug 2008;67(8):1170-3. doi:10.1136/ard.2007.083436

14. Bozzolo EP, Ramirez GA, Bonavida G, et al. Efficacy and toxicity of treatments for nephritis in a series of consecutive lupus patients. *Autoimmunity*. Dec 2013;46(8):537-46. doi:10.3109/08916934.2013.817560

15. Alarfaj AS, Khalil N. Fertility, ovarian failure, and pregnancy outcome in SLE patients treated with intravenous cyclophosphamide in Saudi Arabia. *Clin Rheumatol*. Dec 2014;33(12):1731-6. doi:10.1007/s10067-014-2686-z

16. Kothari R, Digole A, Kamat S, Nandanwar YS, Gokhale Y. Reproductive Health in Systemic Lupus Erythematosus, An experience from Government Hospital in Western India. *J Assoc Physicians India*. Dec 2016;64(12):16-20.

17. Ceccarelli F, Orefice V, Perrone G, et al. Premature ovarian failure in patients affected by systemic lupus erythematosus: a cross-sectional study. *Clin Exp Rheumatol*. May-Jun 2020;38(3):450-454.

**Supplementary Figure S1.**

**
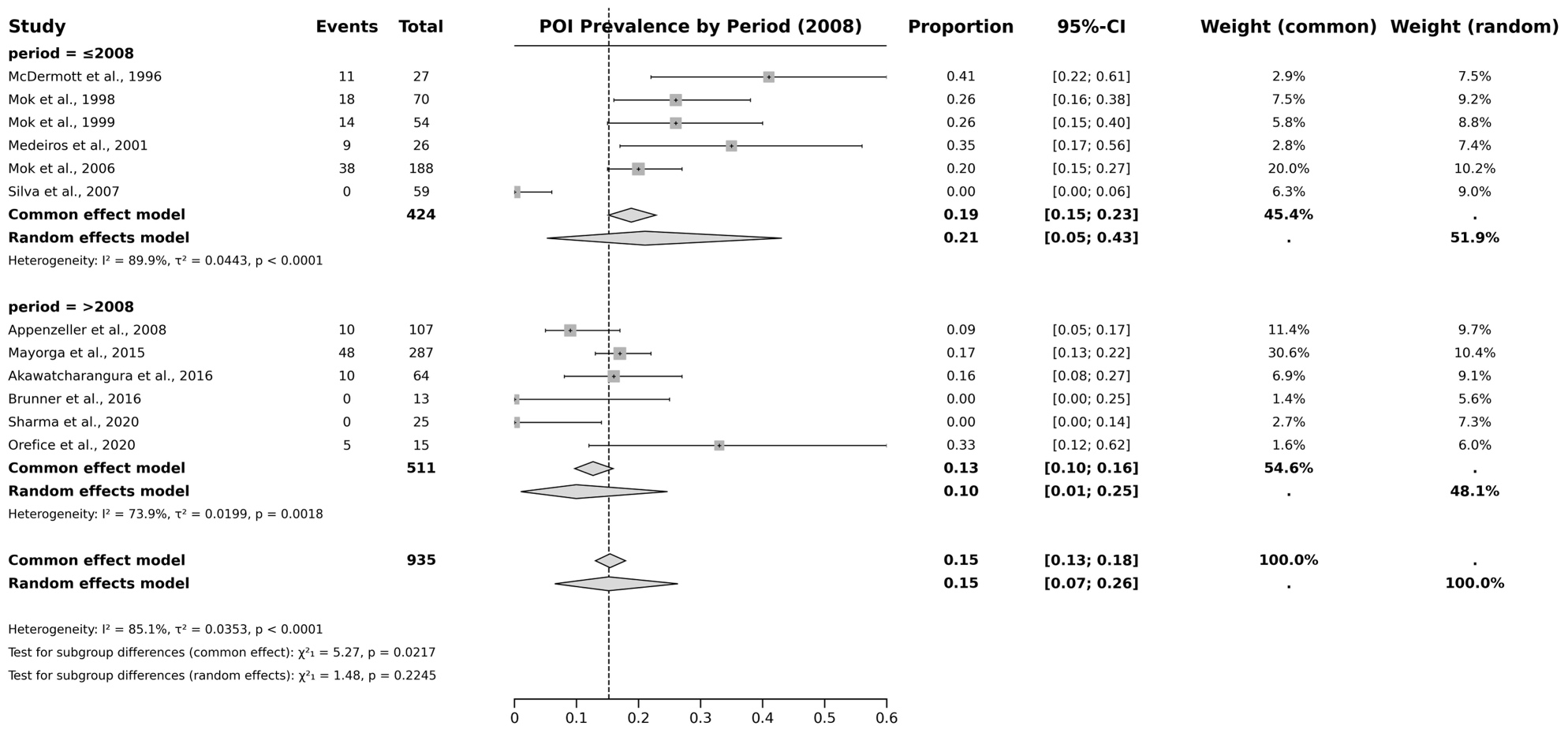
**

**Supplementary Figure S1.** Forest plot of the prevalence of POI in women with SLE treated with CYC, stratified by study period (pre-2008 vs post-2008), The 2008 cut-oint corresponds to the year when the Euro-Lupus low-dose CYC regimen was first cited in the EULAR guidance, although it was not yet part of the formal treatment recommendations. A reduction in POI prevalence was observed in post-2008 studies (10%, 95% CI 1.0-24.7%), supporting a gradual decline associated with earlier adoption of low-dose protocols.

**Supplementary Figure S2.**

**
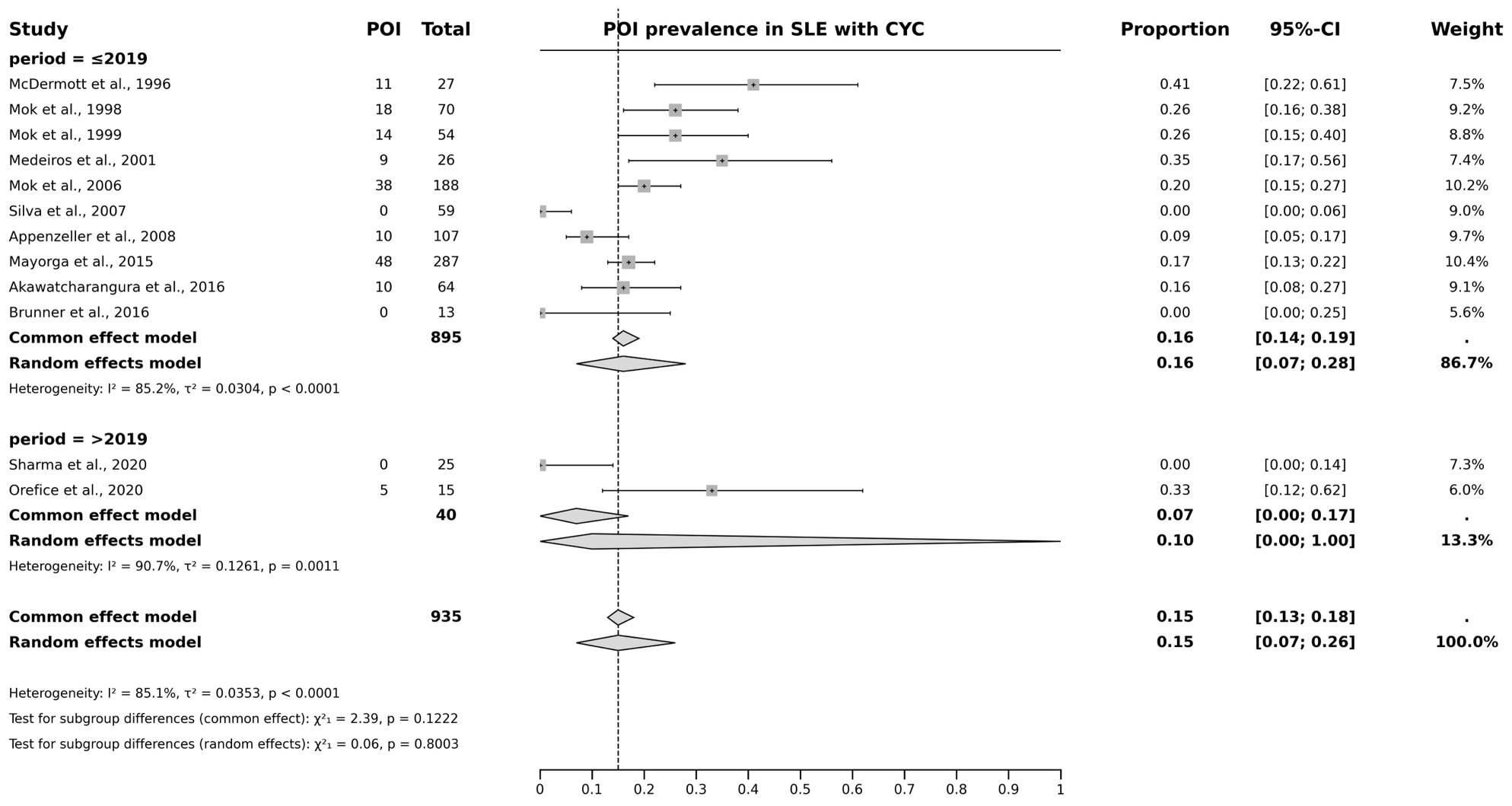
**

**Supplementary Figure S2.** Forest plot of the prevalence of POI in women with SLE treated with CYC, stratified by study period (pre-2019 vs post-2019). The 2019 cut-point corresponds to the publication of the EULAR, which formally endorsed the Euro-Lupus low-dose regimen as a standard induction therapy for lupus nephritis. Only two studies were available in the post-2019 period, yielding very wide confidence intervals (10.3%, 95% CI 0-100%) and non-significant subgroup differences (p = 0.80), indicating that the decline in POI risk predated the official guideline adoption.

**Supplementary Figure S3.**


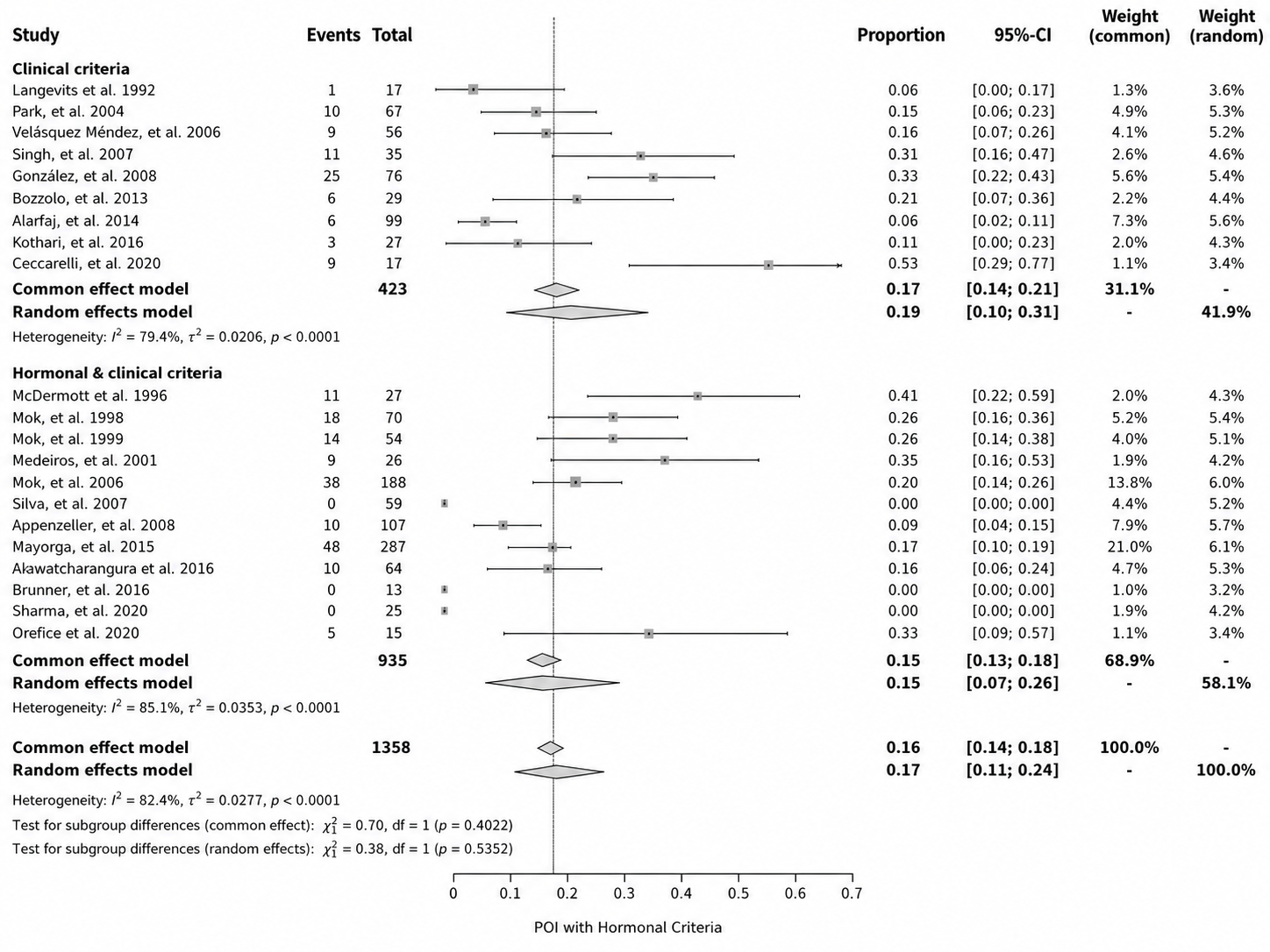


**Supplementary Figure S3.** Forest plot of premature ovarian insufficiency (POI) prevalence stratified by definition criteria. Studies applying strict hormonal definitions (amenorrhea plus abnormal gonadotrophins/estradiol) yielded a pooled prevalence of 15% (95% CI: 7-26%), while those using broader, clinical definitions (sustained amenorrhea) reported a prevalence of 19% (95% CI: 10-31%).

**Supplementary Figure S4.**


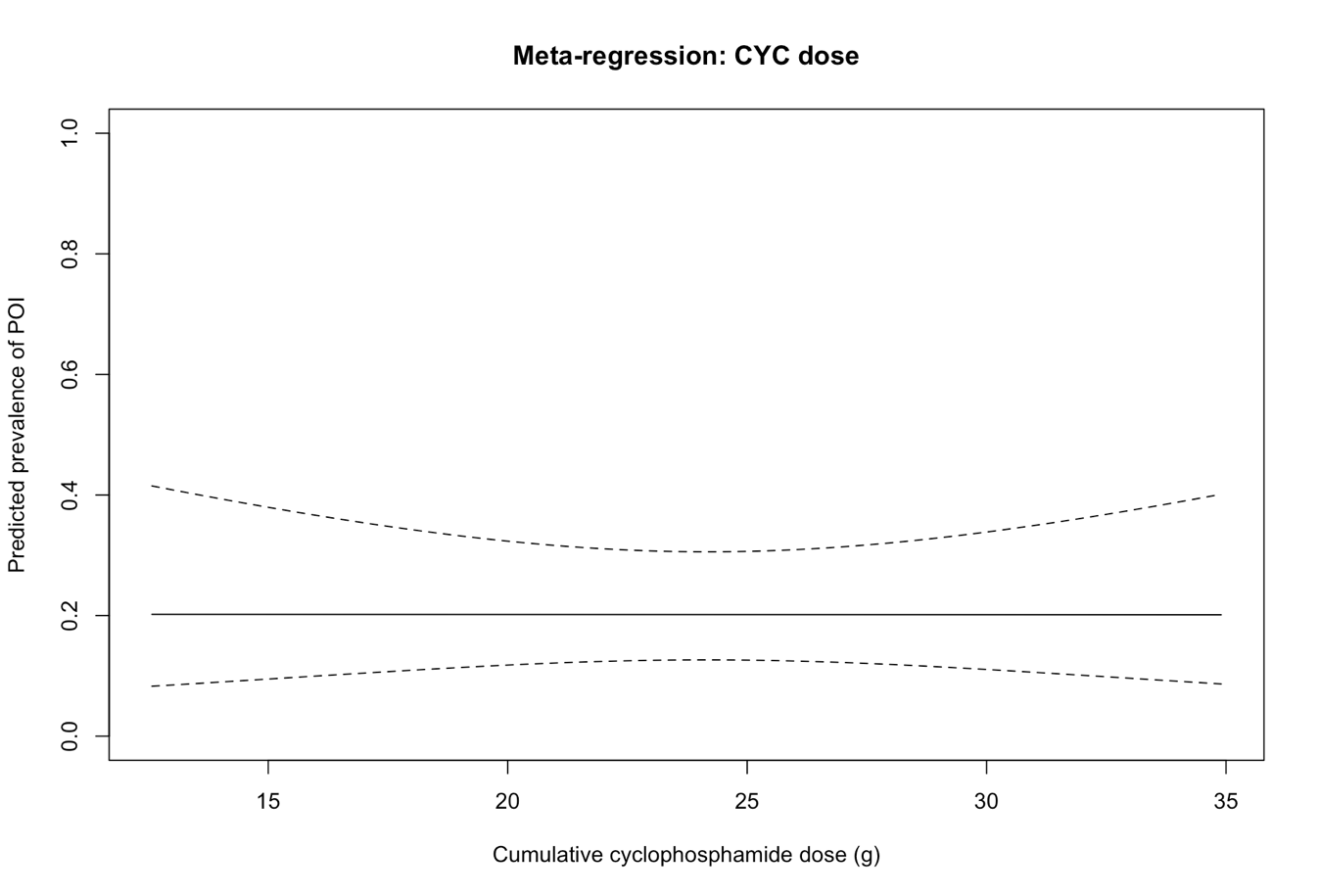


**Supplementary Figure S4.** Meta-regression of cumulative cyclophosphamide dose and premature ovarian insufficiency prevalence, showing no significant association.
